# An Investigation of Mechanical Properties of Recycled Carbon Fiber Reinforced Ultra-High-Performance Concrete

**DOI:** 10.3390/ma16010314

**Published:** 2022-12-29

**Authors:** Andrew Patchen, Stephen Young, Dayakar Penumadu

**Affiliations:** Tickle College of Engineering, The University of Tennessee, Knoxville, TN 37996, USA

**Keywords:** recycled carbon fiber, fiber-reinforced concrete, ultra-high performance concrete, micro X-ray computed tomography, mechanical properties

## Abstract

Carbon fiber-reinforced concrete as a structural material is attractive for civil infrastructure because of its light weight, high strength, and resistance to corrosion. Ultra-high performance concrete, possessing excellent mechanical properties, utilizes randomly oriented one-inch long steel fibers that are 200 microns in diameter, increasing the concrete’s strength and durability, where steel fibers carry the tensile stress within the concrete similar to traditional rebar reinforcement and provide ductility. Virgin carbon fiber remains a market entry barrier for the high-volume production of fiber-reinforced concrete mix designs. In this research, the use of recycled carbon fiber to produce ultra-high-performance concrete is demonstrated for the first time. Recycled carbon fibers are a promising solution to mitigate costs and increase sustainability while retaining attractive mechanical properties as a reinforcement for concrete. A comprehensive study of process structure–properties relationships is conducted in this study for the use of recycled carbon fibers in ultra-high performance concrete. Factors such as pore formation and poor fiber distribution that can significantly affect its mechanical properties are evaluated. A mix design consisting of recycled carbon fiber and ultra-high-performance concrete was evaluated for mechanical properties and compared to an aerospace-grade and low-cost commercial carbon fiber with the same mix design. Additionally, the microstructure of concrete samples is evaluated non-destructively using high-resolution micro X-ray computed tomography to obtain 3D quantitative spatial pore size distribution information and fiber clumping. This study examines the compression, tension, and flexural properties of recycled carbon fibers reinforced concrete considering the microstructure of the concrete resulting from fiber dispersion.

## 1. Introduction

### 1.1. Fiber-Reinforced Concrete Overview

Concrete is the most widely used building material in the world and plays a critical role in the world’s infrastructure [1,2]. Traditionally, concrete has been reinforced with continuous bars for both crack control and tensile load carrying [3]. The cost of continuous reinforced bars and the labor to place them make up a significant part of the cost of concrete construction [4]. One potential solution for this is Fiber-Reinforced Concrete (FRC), where short, chopped fibers are mixed into the concrete to serve and reinforcement [5]. FRC is ideal for use in many applications, such as 3D printing, architectural design, blast protection, and many others [6,7,8,9,10]. 

Various fiber types, including steel fiber, glass fiber, basalt fiber, and synthetic fibers, have been used as reinforcements in concrete [5,10,11]. Steel fiber is one of the most common fibers used in concrete applications and has been demonstrated to increase tensile, compressive, and flexural strength, as well as energy absorption and toughness for FRC [10,12,13,14,15,16]. Additionally, steel fiber exhibits uniform dispersion and suitable workability controlled up to a 3% volume fraction [17]. Furthermore, many studies have been performed using carbon fiber as a reinforcement in concrete [10,18,19,20,21,22,23]. Carbon fiber could prove superior to steel fiber for use in concrete applications due to its better mechanical properties and high corrosion resistance or chemical inertness [18].

### 1.2. Carbon Fiber Advantages

Carbon fiber has a higher modulus than steel, which allows it to develop higher stresses before concrete fails at relatively low tensile strains [24]. Similarly, carbon fiber is chemically inert and does not have the same corrosion issues to which steel fibers are susceptible. This is particularly important in concrete applications because concrete is highly alkaline and potentially used in harsh environments [19]. Carbon fiber’s physical properties could make it an ideal replacement for steel fiber in concrete. However, the price of the fibers plays a significant role in the final cost of the concrete mix [25,26]. This can potentially place carbon fiber at a significant disadvantage compared to steel [27]. One potential new solution to this issue is to use Recycled Carbon Fiber (rCF), which can have a lower cost than virgin fibers [28,29,30].

### 1.3. Recycled Carbon Fiber Overview

rCF is a promising viable option because carbon waste from the automotive and aerospace industries can be utilized [31]. Carbon fiber can be recycled through three processes: mechanical, chemical, and thermal [31]. Despite carbon fiber uses in cement- and concrete-based studies, carbon fiber-reinforced polymers (CFRP) are difficult to recycle, and when burned can sometimes produce toxic fumes. This is due to the thermal and chemical stability of CFRP. Thus, it is critical to develop a recycling method to mitigate environmentally harmful CFRP waste. Furthermore, recycling CFRP is potentially sustainable, reducing the accumulation of CFRP in landfills [32,33]. The process of recycling rCF presents challenges, including fiber damage, fiber length variation, fiber diameter change, fiber contamination, and loss of strength [29,31,34,35,36,37,38,39,40,41,42,43,44,45,46,47]. 

Numerous studies have been performed on rCF-reinforced concrete and cement-based applications [32,48,49,50,51,52]. Ogi et al. used recycled and crushed CFRP pieces to reinforce concrete and investigated compressive and flexural strength and fracture behavior [53]. The adhesion strength of the interface between CFRP and cement was also evaluated by performing pull-out and peel tests [53]. Mastali et al. investigated the use of recycled CFRP for self-compacting concrete for its impact resistance and mechanical properties [54,55]. The study revealed improved compressive strength, flexural strength, and impact resistance for plain self-compacting concrete reinforced using recycled CFRP [54]. However, although increased fiber length and fiber volume fraction enhanced the impact resistance and mechanical properties, the workability of the reinforced mix compositions was reduced [55]. Carbon fiber has been reported for use in smart concrete, such as self-sensing and self-healing concrete, due to its electrical conductivity properties [56,57]. Faneca et al. used polyacrylonitrile (PAN)-based rCF for the development of multifunctional conductive cementitious materials in the civil engineering industry [48]. 

Akbar et al. investigated microstructural milled rCF using scanning electron microscopy (SEM) coupled with energy x-ray dispersive spectroscopy (EDS), thermogravimetric analysis (TGA), x-ray diffraction (XRD), and Fourier transform infrared spectroscopy (FTIR) [32]. The surface defects and grooves provided nucleation sites and better bonding to the cement matrix. XRD, TGA, and FTIR data revealed that hydration products were promoted with these nucleation sites on the rCF. The milled rCF mixed with cement paste was also evaluated for flexural and compressive strength properties compared to plain cement paste. The milled rCF was observed to be uniformly dispersed within the paste. Furthermore, an increase in flexural and compressive strength was observed with the addition of 1% by the volume of milled RCF [32].

### 1.4. Ultra-High Performance Concrete Overview

One of the major applications of FRC is Ultra-High-Performance Concrete (UHPC) [58]. UHPC is defined as having a minimum compressive strength of 124 MPa [59]. It exhibits excellent strength and durability properties and is, therefore, an attractive material for civil engineering applications, including bridges, railway engineering, and construction [12,13,60,61,62,63,64]. UHPC affords excellent rheological properties and workability, along with reduced porosity, in part due to its high cement content, mineral admixtures, and close packing of its constituents [64,65,66]. However, the mix composition, curing conditions, and incorporation of fibers can significantly affect the mechanical properties of UHPC. It has been reported that the inclusion of fibers can increase the mechanical properties of UHPC [67,68]. From a rheological perspective, the fiber geometry, as a needle-like particle, promotes increased possibility of interlocking of fibers and mechanical interaction between the fiber and solid materials [69]. Furthermore, higher amounts of entrapped air in the mixture can result from reduced workability and rheology [70]. However, UHPC has an initial high-cost barrier that is increased by expensive fiber reinforcement, which can affect the practicality of the material [25,26,71]. Yu et al. and Randl et al. investigated the ecological impact of UHPC mix designs by substituting cement with eco-friendly cement and less energy-intensive additives [72,73]. 

One of the key advantages of UHPC is that its tensile strength is significantly higher than that of normal concrete [74]. Fiber reinforcement of the UHPC “results in post-cracking strength retention and a level of ductility uncharacteristic of conventional concrete” [75]. Shafiefer et al. investigated UHPC tensile strength, and ductility was two to four times greater than conventional concrete [74]. The role of tensile strength is typically not emphasized compared to compression and flexural strength due to concrete not typically being designed for direct tension. However, tensile strength is critical for estimating the load conditions that can occur and increase pre-cracking strength [74].

Both carbon fiber and steel fibers have been studied for use in UHPC as reinforcement [76,77]. Similar to steel fiber, the presence of carbon fiber can bridge microcracks in UHPC, allowing the material to continue to carry the load after cracking due to the high bond strength between the carbon fiber and the cementitious-based material [78,79]. Factors such as fiber aspect ratio, length, and geometric shape affect the mechanical integrity of the resulting fiber-reinforced UHPC [12]. Furthermore, the pore structure and porosity distribution can significantly influence the strength of cement-based material [64]. Mercury intrusion porosity (MIP) has been extensively used to evaluate the pore size and microstructure of different materials, including UHPC [80,81,82]. Rios et al. evaluated the tensile properties of steel fiber-reinforced ultra-high strength fiber-reinforced concrete using MIP and X-ray tomography [82]. MIP porosity analysis was demonstrated to be useful for small pores on a scale between 0.003 and 30 μm. The compressive strength of concrete reinforced with steel fibers increased with respect to neat concrete, in proportion to the reduction in its porosity [82].

### 1.5. Micro X-ray Computed Tomography (μ-XCT)

One method for evaluating the pore structure of UHPC is to utilize μ-XCT. μ-XCT is an invaluable non-destructive evaluation method for composite materials with increasingly complex geometry. There is growing interest in examining the microstructure of fiber-reinforced concrete materials. The key benefit of μ-XCT is the ability to evaluate in 3D spatially critical parameters, including cracks, fiber orientation, the bond between fiber and matrix, and porosity, which can affect the mechanical performance of FR concrete [82,83,84,85,86,87,88]. Gao et al. utilized X-ray tomography to identify the carbon fiber distribution morphology of cement-based composites [89,90]. Thus, there is a critical need for a comprehensive structure–property relationship for rCF-reinforced concrete.

### 1.6. Significance of This Research

In this study, the authors developed a multiscale experimental program to evaluate rCF-reinforced UHPC (rCF-UHPC) compared to two commercially available carbon fiber and steel fibers for mechanical properties, including compression and flexural and tensile strength. The carbon fiber types investigated included an aerospace-grade carbon fiber made by Hexcel and an industrial-grade carbon fiber made by Zoltek, along with the rCF provided by Carbon Fiber Recycling LLC. There are limited studies on rCF and its use in UPHC; therefore, it is critical to investigate how it performs in UHPC and how it compares to other commercially available fiber types to determine its viability as a reinforcement for UHPC.

To this end, different carbon fiber types were investigated using TGA to assess the thermal stability and sizing content of the fibers. Scanning electron microscopy was used to analyze the surface morphology of the fibers, and energy X-ray dispersive spectroscopy was used to identify the elemental chemical composition of the fibers. This allowed the different fiber types to be analyzed and showed how rCF compared to the commercially available carbon fiber types physically and chemically. A μ-XCT technique was used to analyze pore geometry and pore size spatially in 3D for each of the UHPC mixes to investigate porosity effects in relation to the mechanical performance of each mixture. The microstructure properties were compared with the mechanical properties and evaluated to show how the pore structure of the different mixes affected the strength of the UHPC mixes. Scanning electron microscopy was also used to image the failed surface of the tensile samples and to provide insight into the failure mechanisms of the concrete and fibers. Overall, this study provides novel insights into how rCF compares to other fiber types, how rCF can be used in UHPC, how μ-XCT can be utilized to show how the microstructure affects the final UHPC mix performance, and how the fibers affect the failure mechanisms of the concrete.

## 2. Materials and Methods

### 2.1. Experimental Program

The multiscale analysis of the concrete mixes used in this study was characterized in three stages. The first stage consisted of characterizing the carbon fiber types for thermal and physical properties. The carbon fibers and steel fiber types were examined using scanning electron microscopy and X-ray energy dispersive spectroscopy to identify the chemical elemental composition of each fiber type. The second stage involved a non-destructive evaluation of the concrete mixes using μ-XCT to study the effect of void volume and size distribution spatially. We also investigated the effects of void formation from the added fiber volume content in the concrete mix, including the resulting reinforcement mechanisms of the fibers surrounded by the host concrete matrix. The third stage evaluated the concrete mix designs for compressive, tensile and flexural strength properties, correlating the porosity and microstructure of the fiber-reinforced concrete mixes.

### 2.2. Concrete Mix Design

Table 1 summarizes the mix design used in the study, where a pre-mix (Ductal’s dark gray UHPC JS1000 premix, Chicago, IL, USA) is combined with a High-Range Water Reducer (HRWR) agent (CHRYSO^®^Fluid Premia 150, Royse City, TX, USA), water, and steel fibers (Ductal Steel Fibers for Ultra-High Performance Concrete (UHPC), Chicago, IL, USA) in accordance with ASTM A820 [91]. According to Ductal, the mix has a high compressive strength, ranging from 100 to 200 MPa, depending on the mix design, and excellent durability and resistance [92]. These properties make it an ideal material for bridges, seismic columns, connections, wind turbine towers, architectural details, and many other uses [92,93]. The premix consisted of cement, sand, ground quartz, and silica flume [94]. The CHRYSO^®^Fluid Premia 150 is particularly useful for UHPC due to its high flowability characteristics, according to CHRYSO [95,96]. The volumes of carbon fiber and steel fiber were kept the same, and the mass used was adjusted based on the fiber’s density. Carbon fibers were not used in the neat concrete mix.

### 2.3. Fiber Types and Properties

**Figure 1:**
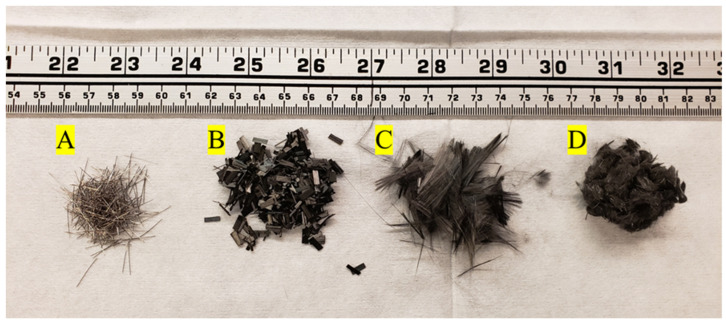
Different fiber types mixed into concrete. (**A**) steel fibers; (**B**) Hexcel carbon fiber platelets; (**C**) Zoltek carbon fiber; (**D**) recycled carbon fiber.

The properties and corresponding images of the fiber types used in this study are summarized in Table 2 and Figure 1, respectively. First, commercially available 6.35 mm chopped aerospace-grade carbon fiber platelets (HexTow^®^ AS/1925 Type 2, West Valley City, UT, USA), representing the high-end of the carbon fiber market, were used for concrete mix design, where the fibers were sized 3–7% by weight with a 1925 sizing system [94,97,98]. As shown in Figure 1B, the sizing of the Hexcel carbon fiber adheres to the fibers into clumps that need to be broken apart during mixing. Second, a commercially available unsized intermediate modulus low-cost continuous carbon fiber 50 K tow (Zoltek PX35, Bridgeton, MO, USA), representing the mid-range of the carbon market, was used [79]. Hexcel carbon fibers are considered in this study due to their commercial popularity for use in chopped fiber carbon composite applications and chemical sizing that is stated by the manufacturer to be compatible as a universal sizing for a variety of composites. As shown in Figure 1C, the Zoltek carbon fibers were sized using a proprietary ammoniated dispersion sizing method appropriate for concrete applications (Michem Prime 4983-40R, Cincinnati, OH, USA), with a targeted sizing of 2% wt, at Izumi International in Greenville, South Carolina, USA [79]. The fibers were then chopped to a nominal fiber length of 12.7 mm using a fiber-chopping module (Cygnet Texkimp Chopping Module, Wincham, Cheshire, UK). The rCFs were supplied by Carbon Fiber Recycling (Tazewell, TN, USA) and used as received as shown in Figure 1D. The rCFs were recycled using a proprietary pyrolysis process. The tensile modulus and tensile strength of the rCF were not measured due to significant variations in fiber lengths and small gauge lengths. However, the length and diameter of the rCF were imaged using a digital microscope (Keyence VHX-7000, Itasca, IL, USA). Seventy rCF fibers were measured for length, and the diameter of the fibers was measured 38 times using the captured optical images and image analysis software (ImageJ, https://imagej.nih.gov/ij/index.html (accessed on 14 July 2022)).

### 2.4. Carbon Fiber Density Measurements

The densities (Table 2) of the three carbon fiber types were measured using a gas pycnometer (Micromeritics, AccuPyc II 1340, Norcross, GA, USA), where 10 samples for each fiber type were measured in a 3.5 cm^3^ cell volume in a helium gas atmosphere. Five cycles were repeated per sample for accuracy and to obtain statistical density values.

### 2.5. Thermogravimetric Analysis of Carbon Fibers

Thermogravimetric analysis (TGA) was performed for the carbon fiber types to observe the degradation behavior and thermal stability of the fibers using a TA Instruments TGA (Q-50, New Castle, DE, USA). Samples for each fiber type with an approximate mass of 8.76 to 16.6 mg were placed in a 100-microliter platinum pan and heated from room temperature to 900 °C at a heating rate of 10 °C/min under an air atmosphere. To calculate the fiber sizing content, isothermal TGA was performed for the carbon fiber types, where samples with an approximate mass of 10.82 to 22.2 mg were heated from room temperature to 400 °C at 10 °C/min and held at 400 °C for 30 min.

### 2.6. Scanning Electron Microscopy and Energy X-ray Dispersive Spectroscopy (EDS) of Carbon Fibers

The surface topography and morphology of the carbon fiber types were observed using a dual-beam scanning electron microscope (SEM) and a Focus Ion Beam (FIB) using a Zeiss Auriga SEM/FIB instrument (Carl Zeiss Auriga^®^ series, Oberkochen, Germany). The fiber samples were Au sputter-coated for 40 s using an Au sputter coater. Additionally, energy X-ray dispersive spectroscopy (EDS) was performed on Au sputter-coated fiber samples using a ThermoFisher Scientific Apreo S SEM (ThermoFisher Scientific, Waltham, MA, USA) with an accelerating voltage of 20 kV to observe the chemical composition of the fibers. The surface topologies of the mechanically failed tensile briquette samples were imaged using an environmental scanning electron microscope (Zeiss SEM EVO^®^ MA15, Carl Zeiss, Oberkochen, Germany) with an accelerating voltage of 5 kV and a secondary electron detector.

### 2.7. Unreinforced Mix and Fiber-Reinforced Mix

The concrete was mixed using the recommended procedure provided by Ductal [100]. A 7.6l Hobert tabletop paddle mixer at medium speed was used to mix the concrete. First, the dry premix constituent was mixed for 2 min. The water and HRWR were added and mixed for approximately 8–10 min. The mixture initially looks dry and typically does not begin to liquefy until about 7 min after mixing once the HRWR and water have been thoroughly mixed together with the premix. By the end of 10 min, the mix was fluid and self-consolidating. The fibers were then gradually added to the mix in small handfuls into the paddle mixer to ensure the consistent mixing of constituents. Once all the fibers were added to the concrete, the mix design was mixed for a further 5 min. Note that carbon fibers tend to clump more than steel fibers, which can lead to issues during mixing. The final mix should be self-consolidating and fluid. To check the mix, a flow test in accordance with ASTM C1437-20 was used to evaluate the rheology of the concrete. The concrete was then ready to be molded into compression cubes, beams, and tension briquettes.

### 2.8. Tensile, Compression, and Flexural Specimen Preparation

**Figure 2:**
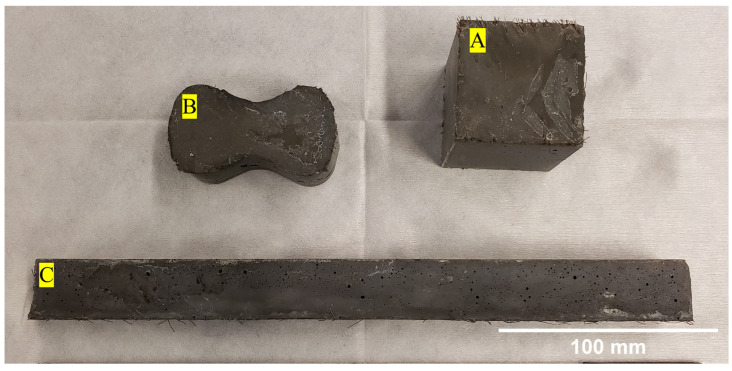
Different sample types used for mechanical testing. (**A**) compression cube (50 mm × 50 mm × 50 mm); (**B**) tension briquettes (at gage 25 mm × 25 mm); (**C**) flexural beam (25 mm × 25 mm × 305 mm).

To properly cast the tension briquettes and compression cubes, two layers of concrete were applied to the steel and brass molds. After each layer, the concrete was tamped 20 times with a 10 mm by 25 mm wooden tamper that compacted and consolidated the concrete. After each layer, the concrete molds were tapped on each side of the mold with a rubber mallet to help release any remaining air pockets. The beam samples were prepared in a similar way, with the exception that instead of being tamped 20 times, the samples were instead tamped such that the entire surface of the sample was tamped at least once. Care was taken for each sample to ensure that the final surface was smooth by using a straight edge to screen off any excess concrete. All samples were cured in molds for 24 h. The molds were then removed, and the samples were transferred to an 80–90% humidity concrete cure room, where the samples were cured for an additional six days for a total curing time of seven days. The seven-day cure time was selected to expedite the testing procedure and allow more rapid iteration. Additionally, due to the nature of UHPC applications, early-setting attributes are often preferred, and we used seven-day strength properties as a reference in this study. Figure 2 shows examples of a tension briquette, compression cube, and flexural beam after molding and curing.

### 2.9. Compressive, Tensile, and Flexural Strengths

**Figure 3:**
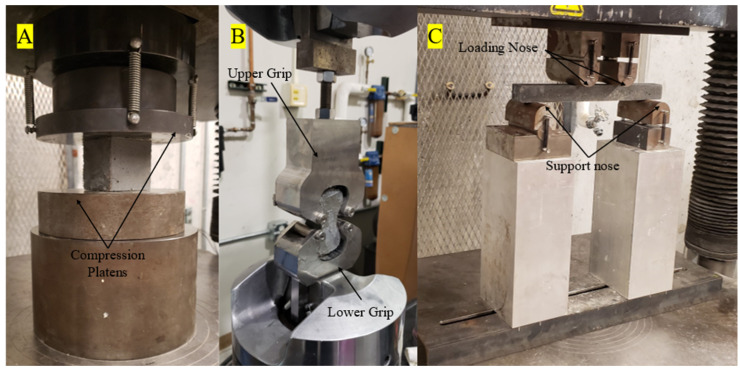
The three different test methods being performed. (**A**) compression cube; (**B**) tensile test; (**C**) flexural test with a 229 mm major span and a 76 mm minor span.

The casted samples were evaluated for their tensile, compression, and flexural strength properties. The compression cubes were tested in accordance with ASTM C109, and the experimental setup is shown in Figure 3A. The samples were monotonically loaded using a 600 kN Instron load frame to mechanical failure. Based on the ASTM C109 standard, the samples should be loaded at a crosshead rate of 900–1800 N/s; however, due to the load frame possessing insufficient force control, it was determined using a displacement control at a rate of 0.1 mm/min, providing consistent results in line with the required force rate [101]. As shown in Figure 3B, the tensile test was performed in accordance with ASTM C307. The tension briquette samples were mounted into custom-made grips using ASTM specifications and loaded monotonically at a crosshead rate of 2 mm/min on a 44 kN MTS servo-hydraulic load frame until mechanical failure [102]. The flexural samples were tested in accordance with ASTM C947 on a 44 kN MTS servohydraulic testing load frame using a custom-made fixture that complied with the standard, as shown in Figure 3C. The samples were loaded monotonically at a crosshead rate of 1.27 mm/min to mechanical failure [103]. It must be noted that a deviation from the ASTM C947 standard was that 25 × 25 × 305 mm samples were cast as previously described, instead of being cut from a sheet of concrete. The force and displacement rates for all cast samples were collected at 5 Hz [103].

### 2.10. Micro X-ray Computed Tomography of Fiber-Reinforced Concrete Designs

Five samples, approximately 18 mm in diameter × 25 mm in length, cored from tensile briquette samples, were scanned using a custom-developed μ-XCT machine. The samples were mounted onto a four-axis (x, y, z, θ) rotary stage, where the samples were scanned using a voltage of 150 kV and an amperage of 137 uA (Hamamatsu L8121-03). The 3001 2D projections (12-bit, 2316 × 2316 pixels at 1× binning) were collected over an angular range of 360 degrees at approximately 0.12 degrees per step at a voxel resolution of 13.89 μm. The 2D projections were normalized, and corresponding sinograms from the projections were used to obtain 2D reconstructed slices using reconstruction algorithm software (Octopus 8.9.3, Ghent University). Correction methods to reduce artificial defects, such as beam hardening and ring artifacts, were applied to the collected projections using the above-mentioned reconstruction software [84]. The 2D reconstructed slices were visualized as a 3D volume, and post-data analysis was carried out on these slices using 3D visualization software (ScanIP, Simpleware T-2022.03-SP1). The grayscale values for each 2D reconstructed slice consisted of values between a minimum value of 0 (black pixel value), corresponding to the least dense regions, such as air, and a maximum pixel value of 65,535 (white pixel value), corresponding to the most-dense regions [88]. A cropped volume of 15.93 mm diameter × 15.81 mm length was used to characterize fiber-reinforced concrete samples for porosity. The voxel resolution of the volume was resampled to 0.03 mm to reduce the image processing computation time, and any defect less than 12 voxels was removed as pixel noise from the reconstructed volume. The resolution of 0.03 mm is larger than the diameter of a single carbon fiber as shown in Table 2 making individual fibers difficult to detect, however, larger fiber bundles can be detected using μ-XCT. As a first step to detect the geometry of the constituents within the reconstructed 3D volume, an upper and lower threshold grayscale value was manually image-segmented to create a mask for each of the concrete, pore, and fiber phases. Flood-fill algorithms were then applied to further enhance the building of the voids and concrete geometries. Boolean operations were then applied to subtract overlapping masks to clearly identify the pores and concrete phases [104]. Each 2D reconstructed slice was manually checked for any overlapping masks or phases. With the exception of the steel fiber-reinforced concrete sample, the segmentation of the fiber phase within the carbon fiber-reinforced samples was not 3D visualized in this study due to similar neighboring grayscale values to the concrete. However, example 2D slices of the carbon fiber bundles were detected and are reported in a later section of this study. Thus, the primary objective was to investigate the 3D spatial distribution of air voids in concrete mix designs and to evaluate the microstructure of the concrete samples.

## 3. Results

### 3.1. Surface Morphology and Elemental Analysis of Fibers

**Figure 4:**
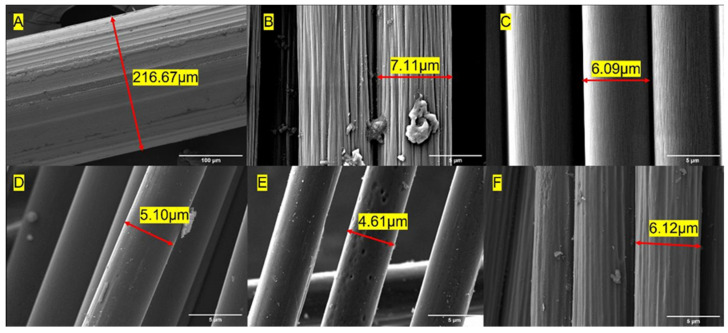
Scanning electron micrographs of (**A**) Steel, (**B**) Zoltek, (**C**) Hexcel, and (**D**–**F**) recycled carbon fibers showing example fiber diameter measurements.

**Figure 5:**
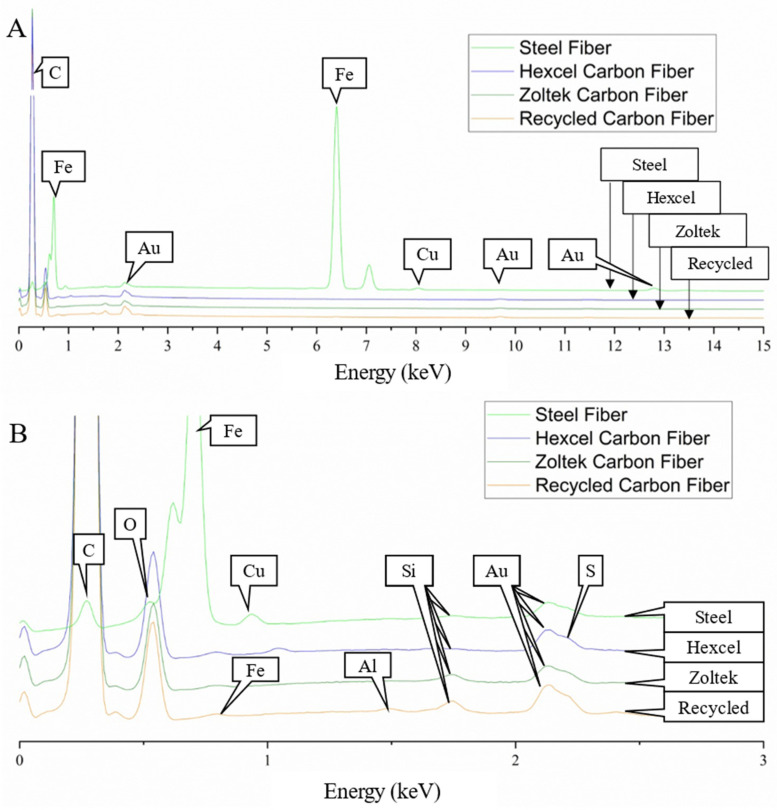
Energy dispersive X-ray spectra of the steel, Hexcel, Zoltek, and recycled carbon fibers, showing the elemental chemical composition of each fiber type. (**A**) Full energy spectrum from 0 keV to 15 keV; and (**B**) inset of energy-dispersive X-ray spectra energy range from 0 to 3 keV for each fiber type. The Au peaks correspond to the Au sputtering of the samples.

Figure 4 shows the fiber surface morphology of the three types of carbon fiber and steel fiber. As shown in Figure 4A, the steel fiber had a rough surface with longitudinal grooves resulting from the drawing process of the manufacturer. The Zoltek carbon fiber (Figure 4B) surface exhibited a rougher surface with clustered regions of sizing compared to the smoother surface exhibited by the Hexcel carbon (Figure 4C). The rCF exhibited a mixture of fibers with rougher and smoother surfaces, as expected, due to the varying sources of carbon fiber material (Figure 4D–F). Zoltek and rCF both have small particles of non-uniform polymer sizing adsorbed on the fiber surface [32]. The resulting surface roughness speaks to the precursor fiber spinning process and subsequent treatment during the conversion process. Trace amounts of residue visible on the rCF surface suggest that fiber sizing played a minimal role during the concrete mixing process. Both Zoltek carbon fiber (Figure 4B) and rCF (Figure 4F) fiber surfaces with rough groove channels suggest improved strength and bonds between the fiber and the hydration paste [49]. Figure 5 shows the energy-dispersive X-ray spectra of the steel, Hexcel, Zoltek, and recycled carbon fibers. Strong C and O peaks were unsurprisingly observed for all three carbon fiber types. The O peaks correspond to the oxidation of precursor fiber complex chemical reactions during the conversion process of precursor fiber to carbon fiber [105]. Trace elements of Si, traditionally used in spin finish oil to protect precursor fibers, were detected for all carbon fiber types [106]. Additionally, the presence of Si suggests fragments from polymer sizing were detected [107]. The detection of O and Si suggests that fibers were exposed to similar chemical elements during the manufacturing process, including precursor, stabilization, and carbonization stages. A sulfur peak was detected for the Hexcel fiber. Fe and Al were detected in the rCF fiber, suggesting that contaminants were present during the thermal treatment process. The steel fiber possessed a Fe, O, Cu, C, and Si chemical composition. However, the resulting final chemical compositions of the carbon fiber and steel fiber were largely dependent on the manufacturer, treatment, and supplier or handling of the fibers.

### 3.2. Thermogravimetric Analysis of Fibers

**Figure 6:**
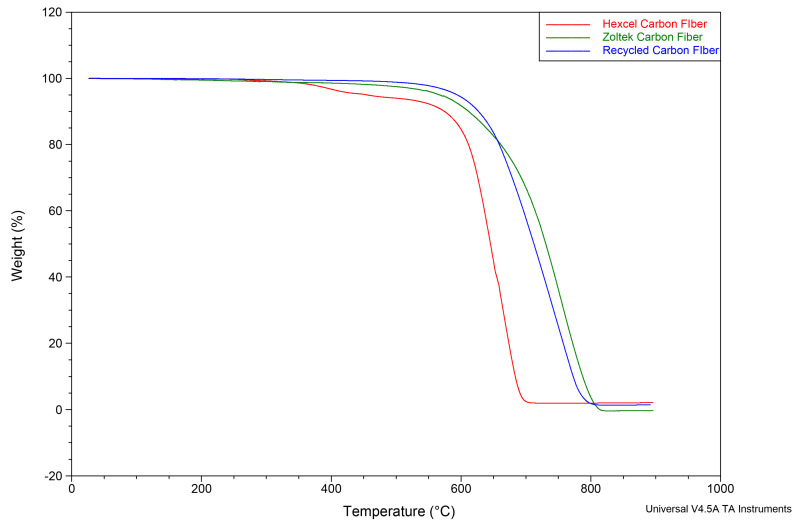
Thermogravimetric analysis curves of Zoltek, Hexcel, and recycled carbon fibers heated in air from room temperature to 900 °C, showing the oxidation degradation of the fibers.

Figure 6 shows a comparison of the thermal stability of oxidation degradation behavior for the three carbon fiber types in an air atmosphere. Two onsets (346 °C and 609 °C) of thermal degradation were observed for the Hexcel carbon fibers compared to the onset for both the Zoltek carbon fiber and the rCF. The rCF and Zoltek carbon fiber TGA curves were comparable, with the rCF (634 °C) having a slightly lower (5%) onset than the Zoltek carbon fiber (667 °C). Table 3 summarizes the thermogravimetric isothermal properties of carbon fibers under a nitrogen atmosphere to evaluate the approximate fiber sizing content for each fiber type. The Hexcel carbon fiber sizing content was 5.91%, which is in the sizing range specified by the manufacturer [98]. Furthermore, the Hexcel carbon fiber sizing content was approximately 143% greater than the rCF sizing content (fiber sizing content = 0.99%). The Zoltek carbon fiber sizing content varied and was approximately 27% (0.75% fiber sizing) to 88% (2.54% fiber sizing) greater than the rCF sizing content.

### 3.3. Physical and Mechanical Properties of Fiber-Reinforced Concrete Mixes

**Figure 7:**
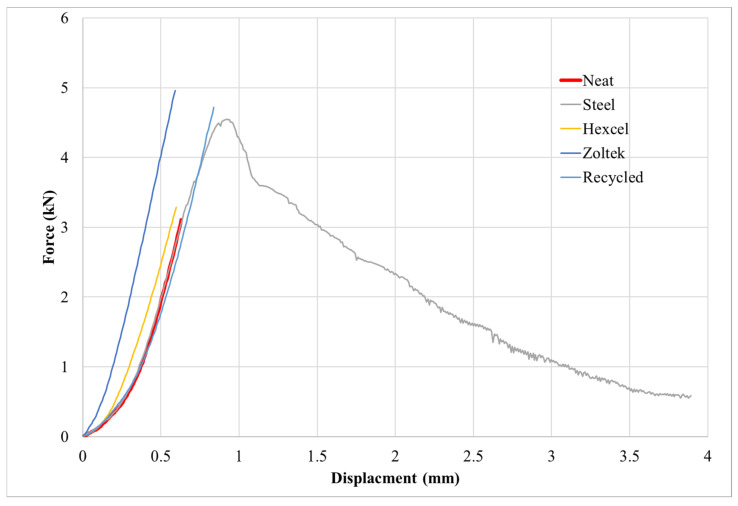
Example tensile force-displacement curves of the briquette concrete samples. Note how the steel fibers continue to carry load after failure compared to the carbon fiber samples that fail, similar to the neat sample.

The neat concrete and steel fiber-based concrete mix designs were used as a baseline to compare the three carbon fiber-based concrete mixtures. Table 4 lists the different mechanical properties, along with their rheology and density. The flow of each mix was recorded, with the neat having the highest at 133%, the steel having the second best at 74%, followed by the Hexcel carbon fiber with 44%, the rCF with 36%, and the Zoltek carbon fiber with 27%. The compressive strength of the rCF mix design (135.3 MPa) was approximately 42.5% higher than the neat mix design (87.9 MPa), approximately 17.9% higher than the steel mix design (113.1 MPa), approximately 0.6% higher than the Hexcel carbon fiber mix design (134.5 MPa), and about 23.2% higher than the Zoltek carbon fiber mix design (107.2 MPa). The tensile strength of the rCF mix design (6.89 MPa) was approximately 27.8% higher than the neat mix design (5.21 MPa), approximately 4.6% higher than the steel mix design (6.58 MPa), approximately 26.3% higher than the Hexcel carbon fiber mix design (5.29 MPa), and approximately 26.4% lower than the Zoltek carbon fiber mix design (8.99 MPa). The flexural strength of the rCF mix design (10.7 MPa) was approximately 8.9% lower than the neat mix design (11.7 MPa), approximately 18.6% lower than the steel fiber mix design (12.9 MPa), approximately 0.9% lower than the Hexcel carbon fiber mix design (10.8 MPa), and approximately 8.5% higher than the Zoltek carbon fiber mix design (9.83 MPa). A basic density measurement of the samples was taken by measuring the sample size with calipers, calculating the volume, and using a scale to measure the mass of the sample. The density of the rCF mix design (2.30 g/cm^3^) was approximately 7.9% less than the neat mix design (2.49 g/cm^3^), approximately 5.1% lower than the steel fiber mix design (2.42 g/cm^3^), approximately 5.8% higher than the Hexcel carbon fiber mix design (2.17 g/cm^3^), and approximately 3.1% higher than the Zoltek carbon fiber (2.23 g/cm^3^). Figure 7 shows the tensile load–displacement curves for each concrete mix design where the curves were based on the load frame displacement measurement. The tensile results for the steel fiber-reinforced concrete mix design demonstrated elastic behavior until the first cracking, with maximum loading occurring at approximately 4.5 kN, with subsequent post-cracking strength indicating fiber pullout failure mode. The carbon fiber and neat concrete mix designs exhibited elastic behavior until catastrophic mechanical failure modes.

### 3.4. X-ray Computed Tomography Results

**Figure 8:**
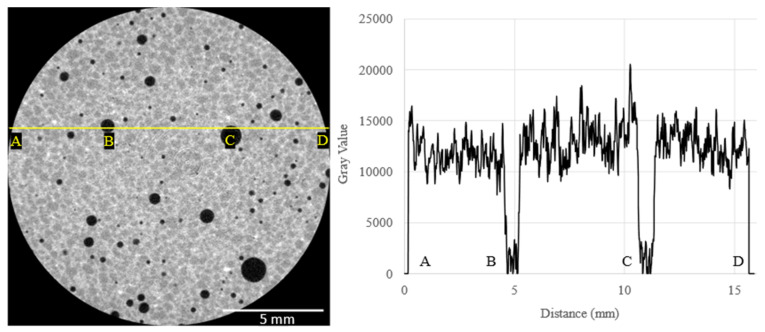
The left image is a 2D reconstructed cross-section of a neat concrete specimen. The right image is the corresponding line profile of grayscale values intensity values based on scale from 0 (black pixel value) to 65, 535 (white pixel value) identifying key microstructure within the concrete mix design designated by points (**A**–**D**). (**B**,**C**) both show porous or air void regions spatially and their corresponding grayscale values.

**Figure 9:**
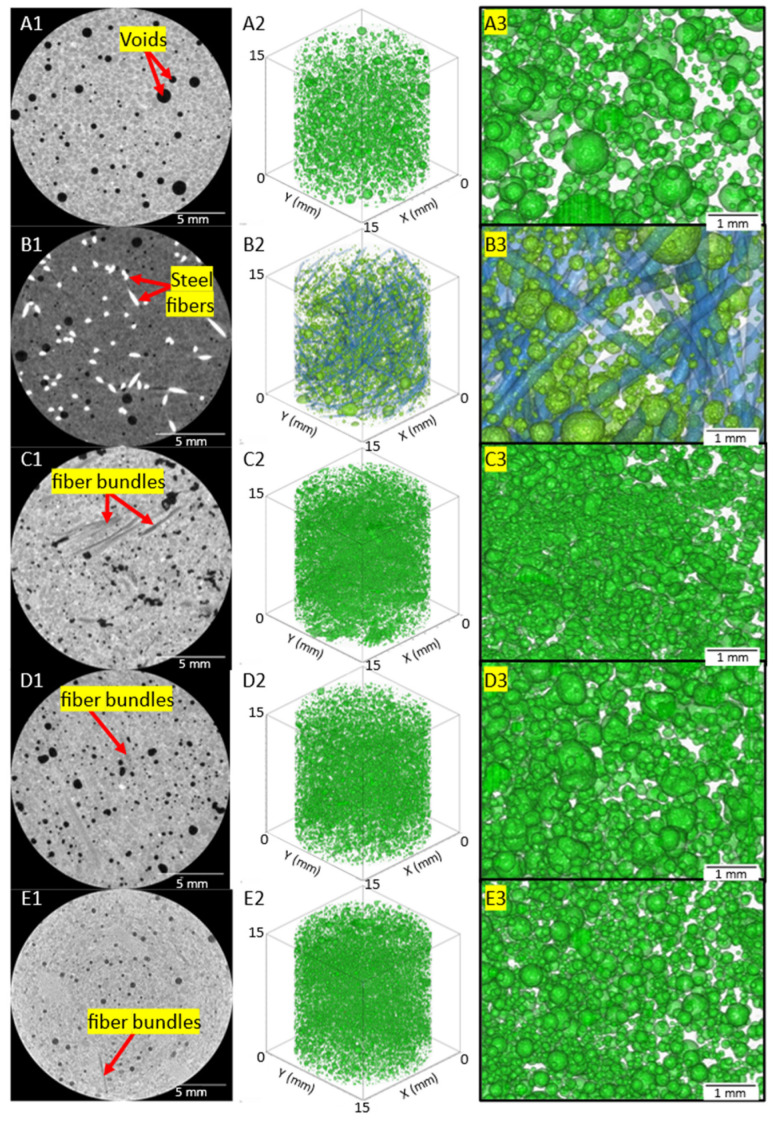
(**A1**–**E1**) 2D reconstructed cross-sections showing air voids, and fiber bundles phases within the host concrete. (**A2**–**E2**, **A3**–**E3**) 3D reconstructed volume showing voids spatially within the concrete volumes. (**A**) neat concrete; (**B**) steel fiber concrete; (**C**) Hexcel carbon fiber concrete; (**D**) Zoltek carbon fiber concrete; (**E**) recycled carbon fiber concrete. Note: For (**B2**) and (**B3**) steel fiber phase is shown.

**Figure 10:**
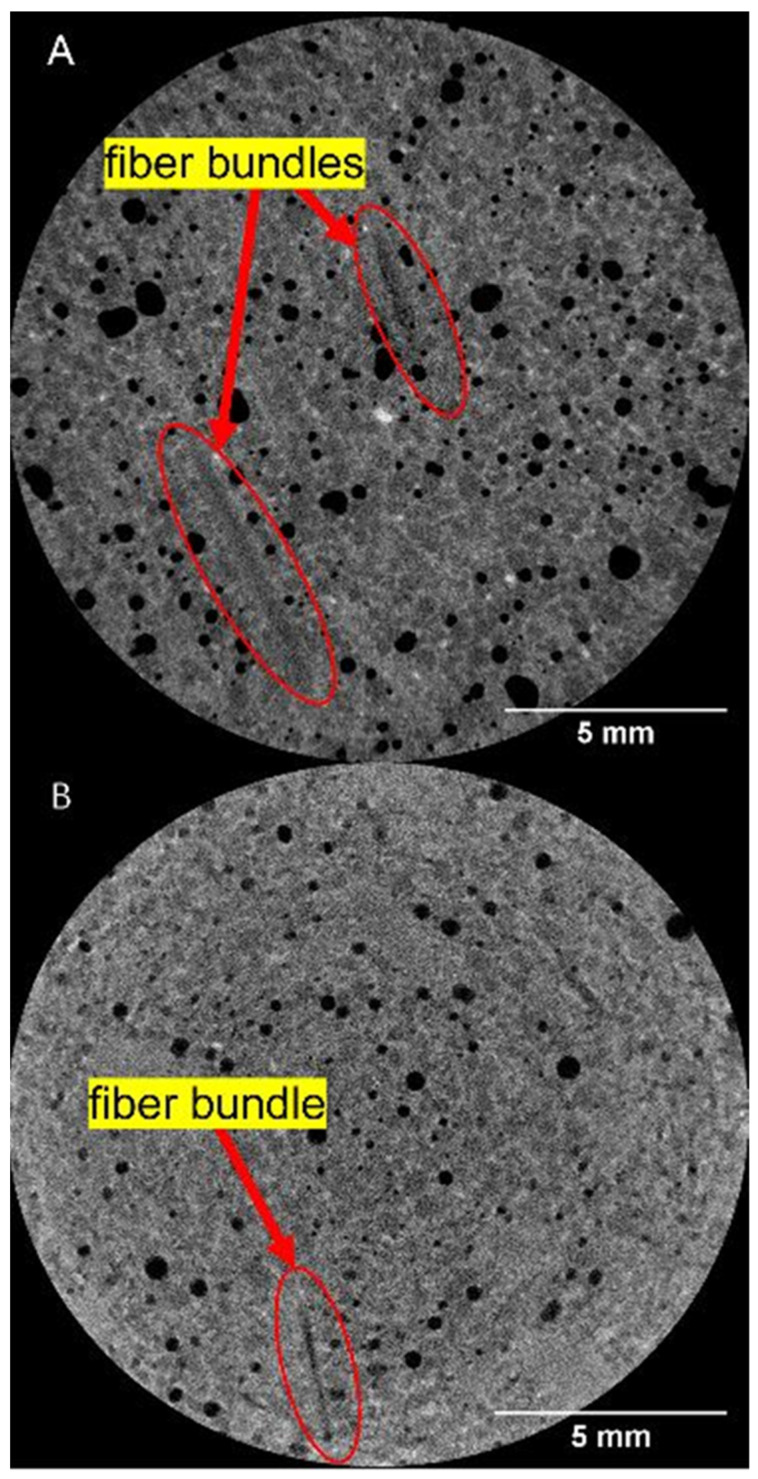
2D reconstructed cross-sections of the (**A**) Zoltek carbon fiber and (**B**) rCF reinforced concrete mix design showing detected fiber bundles’ microstructural features using μ-XCT technique.

**Figure 11:**
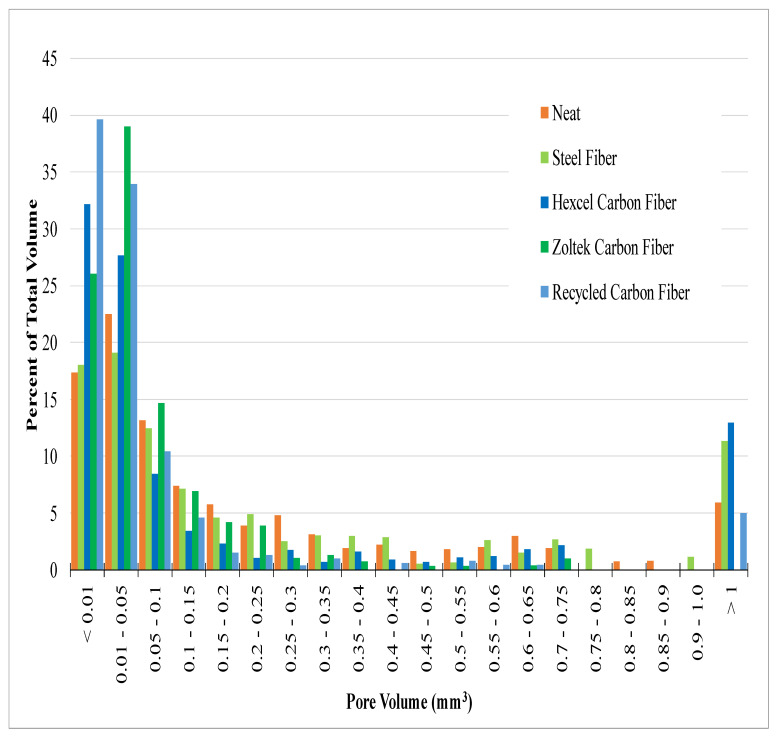
Pore volume distribution with respect to percent of total void volume.

Figure 8 shows an example 2D reconstructed slice of a neat concrete sample with clearly observable concrete and pore phases. The least dense regions, corresponding to the pores, approached zero grayscale intensity values, and the concrete had a gray value of around 12,000. This demonstrates the effectiveness of μ-XCT to segment the pore or air void phase from the concrete for post-processing of scanned samples to quantify pore size distribution. Figure 9 shows examples of 2D reconstructed slices and 3D reconstructed volumes of segmented pores of the cored concrete samples used in this work. The first column in Figure 9A1–E1 shows clearly visible air voids within the 2D reconstructed cross-section for each concrete mix design. In the steel fiber mix design sample (Figure 9B1), the steel fibers were clearly visible due to the attenuation contrast between the concrete and the steel fibers. Due to the significant overlapping of grayscale values between carbon fiber and concrete, the fiber phase did not exhibit a stark contrast between the phases. However, the clumped fiber bundles for the Hexcel carbon fiber sample were clearly visible. The 2D reconstructed cross-sections for Zoltek (Figure 9D1) and rCF fiber (Figure 9E1) suggest that the fibers were more uniformly distributed compared to the Hexcel carbon fiber concrete sample, with fiber bundles shown with elliptical regions, as highlighted in the 2D cross-sections shown in Figure 10. Due to the significant overlap of the grayscale values of the fibers and concrete matrix in the Zoltek carbon fiber and rCF concrete mix designs, image threshold detection of the fiber bundles by the Simpleware software were more subtle compared to Hexcel carbon fiber and steel fiber. The second (A2–E2) and third columns (A3–E3) in Figure 9 show the 3D segmented volume and sub-volume of void phases for each concrete sample, respectively. Table 5 summarizes the void volume fraction, calculated using Simpleware software, in which the total voxels of the void phase were divided by the total concrete volume (fiber, void, concrete). The void volume fractions of the steel fiber and neat mix designs were the lowest, at 2.55% and 3.46%, respectively. The rCF mix design had the lowest void volume of the carbon fiber mixes, followed by Hexcel carbon fiber and then the Zoltek carbon fiber, with values of 3.98%, 4.02%, and 4.38%, respectively. The increased porosity observed with the addition of fibers to the concrete mix design can be attributed to the decrease in the packing density of the carbon fibers compared to steel fibers [69]. The voids observed for the neat, steel, Zoltek carbon fiber, and rCF concrete mix designs exhibited the most spherical geometry, while the Hexcel designs appeared to have non-spherical geometry. This could be attributed to the fiber clumping and poor workability of the Hexcel carbon fiber mix design compared to the other mix designs. Figure 11 shows the void size distribution comparison for the five concrete mix designs used in the study, where the volume void size ranged from 0.01 to 1 mm^3^. The steel fiber and neat concrete had a relatively even distribution of voids compared to the carbon fiber samples, with a peak at voids with a volume of 0.01–0.05 mm^3^. The carbon fiber samples had a higher peak in voids, with a volume below 0.05 mm^3^. There was also a significant concentration of voids greater than 1 mm^3^ in volume observed for the concrete design mix, with the exception of Zoltek carbon fibers, which had no voids larger than 0.75 mm^3^ in volume. The rCF mix had a relatively higher volume of small voids (39.6%), below 0.01 mm^3^, but fewer large voids than the other carbon fiber types.

### 3.5. SEM Failure Images

**Figure 12:**
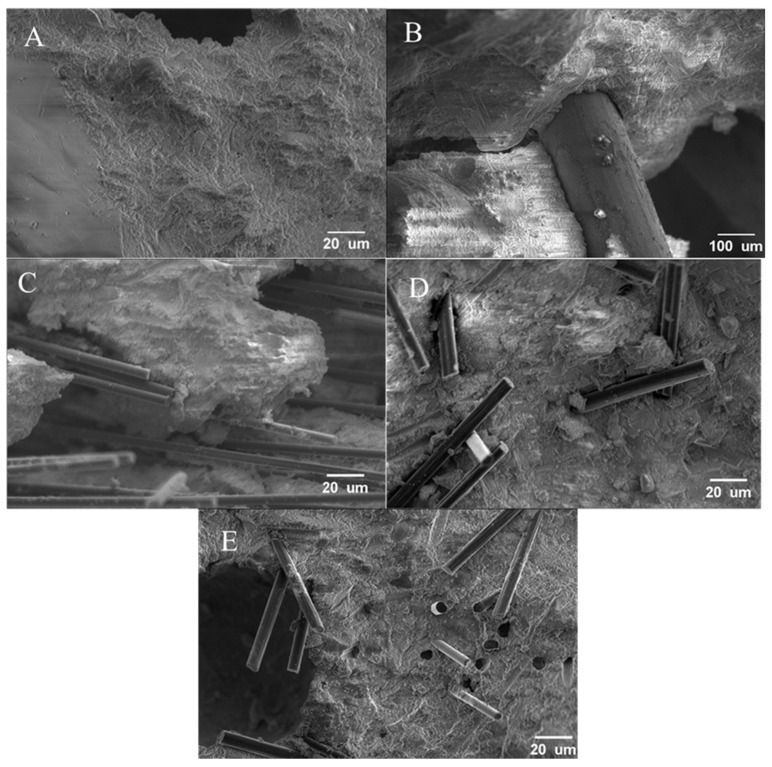
Scanning electron microscopy of mechanically failed (**A**) neat concrete sample; (**B**) steel fiber; (**C**) Hexcel fiber; (**D**) Zoltek fiber; and (**E**) rCF-reinforced tensile briquette samples.

Figure 12 shows an example of SEM micrographs of the tensile samples after mechanical failure. Figure 12B shows that the steel fiber did not rupture; instead, the fiber debonded from the concrete. Further, as shown in Figure 12B, there was minimal concrete bonded to the fiber. The Hexcel (Figure 12C), Zoltek (Figure 12D), and rCF (Figure 12E) carbon fibers exhibited fiber pull-out failure mechanisms. In Figure 12C, the Hexcel carbon fiber ruptured; however, the fibers did significantly pull out of the concrete. The Hexcel fiber bundles also showed a strong fiber orientation and clumped together, indicative of failure initiation zones within the concrete volume. Compared to the other two carbon fiber types (Zoltek carbon fiber and rCF), the Hexcel carbon fiber pull-out was clearly longer, indicating a longer critical fiber length and, therefore, poorer bonding and stress development in the fiber than the other two types of carbon fiber. The Zoltek (Figure 12D) and rCF (Figure 12E) both showed random fiber orientation, suggesting that the fibers were mixed with minimal clumping compared to the Hexcel fiber (Figure 12C). This observation is consistent with XCT 2D reconstructed cross-sections, indicating that μ-XCT is an effective non-destructive technique to spatially detect larger fiber bundles within the concrete mix design. In Figure 12D, the Zoltek carbon fiber concrete mix design shows relatively short fibers pultruding out of the concrete matrix, suggesting that, while the fibers rupture, significant debonding and pull-out mechanical failure behavior is apparent. However, compared to the Hexcel carbon fiber, the Zoltek fibers showed less clumping. Figure 12E shows the rCF mix and, similarly to the Zoltek carbon fiber, pull-out and rupture were observed. The holes for the fiber pull-out of the rCF sample (Figure 12E) from the opposite break face were obvious, showing that, while the fibers were breaking, there was still significant debonding occurring, and the fibers were not developing their full strength.

## 4. Discussion

The primary objectives of the study included performing a multiscale analysis investigating the relationship between rheological behavior, microstructural features of interest, such as porosity, and mechanical performance for all the considered mixes, particularly the influence of carbon fiber reinforcement in the concrete mix design. Considering the inverse relationship between increased fiber volume content and flowability, introducing fiber into the mix reduced the flow, as expected, which can affect the mechanical performance of the specimens [108]. It was therefore theorized that the reason for the reduced mechanical properties compared to the steel fibers was due to the carbon fibers reducing the flow of the concrete, leading to increased air voids and poor fiber dispersion. As shown in Table 4, the flowability of all three carbon fiber reinforced mixes (27–44%) was significantly lower compared to the flowability achieved by steel (74%) or neat concrete (133%). The reduction in flow and poor fiber dispersion of the carbon fiber types can be attributed to three primary factors. First, the sizing on the Hexcel carbon fiber, which does not appear to be chemically compatible with the concrete, led to the fibers clumping together instead of dispersing. Second, the nominal 12.7 mm chopped length of the Zoltek carbon fibers is theorized to have affected the efficiency of fiber dispersion, where the length of the fibers can cause fiber entanglement. In general, shorter fiber lengths can be more easily mixed and dispersed throughout the concrete mix due to fewer fibers tangling with themselves. Recycled carbon fibers with shorter fiber lengths may possess residual polymer sizing, causing them to exhibit poor flowability. Third, for all of the carbon fiber types, the fibers had a much smaller diameter than the steel fibers (200 μm) meaning there are many more individual fibers to disperse and much more surface area that needs to be wet by the concrete.

The reduced flowability affects how the concrete compacts into the mold, leading to increased porosity, void size, and poor dispersion of fibers in the concrete. Increased porosity can be observed through a decrease in the density of the concrete. The carbon fiber-based concrete mixes all have a much lower density (2.17–2.3 g/cm^3^) than the steel (2.42 g/cm^3^) and neat (2.49 g/cm^3^) concrete. This indicates that air voids were trapped in the concrete since the addition of the lighter-density carbon fiber did not change the density of the concrete as much as was observed. Thus, these factors suggest that the mechanical performance of the carbon fiber-reinforced concrete mixes was significantly influenced by increased air voids and poor microstructure, which had a significant influence on the mechanical strength properties (compression, tensile, flexural), negating the full potential benefits of the carbon fiber reinforcement.

Furthermore, the tensile force-displacement curves (Figure 7) showed that the steel fibers provided significant post-peak strength, indicating gradual debonding as the steel fibers were pulled out of the concrete matrix. Conversely, the carbon fiber-reinforced concrete mix design tensile samples all exhibited mechanical behavior similar to the neat concrete mix, with no strength after initial cracking, suggesting that the samples experienced brittle failure. The SEM micrographs (Figure 12) also revealed that the carbon fibers pulled out of the concrete host matrix. However, due to their smaller diameter and poor shear strength across the diameter of the fiber, the fibers were sheared by off-axis loading when a crack formed, preventing any significant post-cracking strength. Therefore, it is clear that steel fibers provide significantly more post-cracking durability than carbon fibers.

The μ-XCT void content results shown in Table 5 positively correlated with the density and flow results (Table 4), with the neat and steel fiber-based concrete having a lower void content than the carbon fiber samples. Utilizing μ-XCT confirmed that the density differences shown were not due to the mass of the fibers used but were instead the result of increased air voids caused by the low flow of the carbon fiber-based concrete mixes. The μ-XCT quantitatively provided void size distribution information for each concrete mix design considered in this study as shown in Figure 11. A clear trend of void volume size was observed, with the majority of the void volume consisting of voids less than 0.1 mm^3^ in size and with another distinct peak of voids greater than 1 mm^3^. Considering the void size distributions (Figure 11) and comparing them with the mechanical performance of each concrete mix design (Table 4), a few key observations can be made. First, while the Zoltek carbon fiber-reinforced concrete mix design had the highest void content, the mix possessed no voids greater than 1 mm^3^ and had the highest tensile strength (8.99 MPa). However, the Hexcel carbon fiber had the highest percentage of void content over 1 mm^3^ (13.0%) and the lowest tensile strength (5.29 MPa) of the fiber-reinforced concrete. This observation suggests that the tensile strength of the concrete mix is not just affected by the strength of the fiber used but also the void content, particularly large voids over 1 mm^3^ (Figure 11). As shown in Figure 11, a similar trend for the rCF and steel fiber mixes was observed, with the steel fibers having a higher percentage of large voids (11.4%) and a slightly lower tensile strength (6.58 MPa), than the rCF mix’s tensile strength (6.89 MPa) and large void percent (5.0%). The comparable tensile strength shows that rCF performs on par with steel fiber and could be a potential replacement for steel fiber in concrete applications. It should be noted that while the neat mix has a low large void content (5.9%), there are also no fibers to transfer the tensile loads, leading to a significant reduction in tensile strength. Thus, it can be theorized that while added fiber reinforcement can increase the tensile strength of concrete, the addition of the fibers decreases the workability significantly and can lead to lower-than-expected strength due to increased porosity and void size, to the point that the benefits of the fiber can be completely negated.

The effect of voids on concrete strength can be further illustrated through other studies that have investigated how void content and void distribution affect the strength of concrete. Li et al. developed a model for how voids affect the tensile and compressive strengths of concrete. In their study, they examined samples with different void contents and void sizes ranging from 0.01 mm to 1.6 mm and found that increasing the total void volume and increasing the size of the voids at the same total volume decreased tensile and compression strength [109]. Knab et al. found that increasing the void content decreased the flexural and compressive strength of concrete [110]. These results correlate well with this study’s test results, which showed that Hexcel carbon fiber samples with both a high void content and large voids performed poorly in tension compared to the other mixes. Further, considering the theory that reduced porosity within the concrete mix yields higher mechanical properties, it was observed that the flexural strength (Table 4) of the concrete mixes correlated almost exactly with porosity values obtained from μ-XCT (Table 5), with the exception of a slight inverse relationship between Hexcel (4.02%) and rCF (3.98%). However, this specific observation of the porosity and flexural strength of the concrete mixes used in this study indicates that porosity and rheology play critical roles in the strength of the concrete. Additional experimental studies on the effects of void content and size distribution resulting from the rheology of the mixes on the strength of the fiber-reinforced concrete mixes, specifically rCF in UHPC, are needed. Nevertheless, the results presented show that μ-XCT is an effective non-destructive evaluation technique for investigating the effects of rheology on the microstructure structure of concrete.

Furthermore, the SEM micrographs (Figure 12) of the failed sample can help explain the differences in the mechanical performance of the fiber-reinforced concrete mix designs. For all fiber types, including steel fiber, the fiber debonded from the concrete instead of having clean breaks. This indicates that the fibers were not able to develop their full strength before they were pulled out of the concrete, with minimal concrete adhering to the fibers. Fiber pulling out and the lack of concrete adhering to the fibers indicate that the fibers did not bond well to the concrete. It is critical that future work take a more in-depth approach to examine fiber–concrete adhesion. Increasing the bond could help to increase the load carried by the fiber and reduce the fiber length needed. The fiber concrete bond can be improved by sizing the fibers and has been shown to improve the bonding with the Zoltek carbon fibers, which showed the highest tensile strength (8.99 MPa). Further, the surface morphology of the fibers can play a role with the rough surfaces of the Zoltek carbon fiber and rCF, providing an additional area for the concrete to bond with the fiber. This is potentially confirmed by observing a higher tensile capacity and reduced pullout in the SEM micrographs.

Based on these characterizations, rCF is comparable to commercially available Zoltek carbon fiber in both chemical composition and thermal degradation. The rCFs mechanical performance was comparable to steel fiber and Zoltek carbon fiber, showing that it is a suitable reinforcement for concrete. The rCF had a lower porosity than the other two carbon fiber types and fewer large voids than the Hexcel carbon fiber. Based on the SEM micrographs of the tensile samples, the rCF had comparable fiber dispersion and fiber bonding to the Zoltek carbon fiber, which was sized for concrete applications. Thus, rCF could serve as a viable replacement for steel fiber as a low-cost carbon fiber alternative in UHPC.

## 5. Conclusions

The effect of using recycled carbon fibers in ultra-high-performance concrete was evaluated for its physical and mechanical properties. The recycled carbon fiber-reinforced concrete design mix was compared to two commercially available carbon fibers, aerospace-grade carbon fiber with platelet geometry and intermediate modulus chopped low-cost carbon fibers. The three carbon fiber reinforcements were evaluated and compared with neat concrete and steel fiber-reinforced concrete. The carbon fibers were evaluated for their flowability in concrete, and physical properties, including density. The carbon fibers and steel fibers were evaluated for thermal stability in air using thermogravimetric analysis. Additionally, the surface morphology of the fibers and elemental chemical composition were examined using scanning electron microscopy, coupled with X-ray dispersive spectroscopy analysis. Micro X-ray computed tomography was used to provide spatial quantitative information on the microstructure of the concrete mix designs used in this study, including void volume size and distribution relationship with the mechanical performances of the resulting concrete mix. The considered concrete mixes were evaluated for tensile, flexural, and compressive properties. Scanning electron microscopy was also used to investigate the failure modes of the tensile samples. The following conclusions can be drawn from the concrete mixes evaluated in this study:

1. The thermal, density, and scanning electron microscopy coupled with energy X-ray dispersive elemental analysis characterizations revealed that the recycled carbon fibers were chemically and physically comparable to Zoltek and Hexcel carbon fibers.
2. The addition of carbon fiber significantly reduced the flow and workability of ultra-high-performance concrete compared to steel fiber and neat concrete mix, leading to increased porosity.
3. The addition of recycled carbon fibers can increase the tensile and compressive strength of ultra-high-performance concrete and is comparable to steel fibers.
4. Micro X-ray computed tomography is an effective tool for the non-destructive evaluation of concrete mix design to obtain quantitative spatial information on microstructural features of interest, including formation porosity, void size distribution, and potential fiber clumping.
5. All fiber types of reinforced concrete mix designs indicated fiber pull-out as the dominant failure mechanism for the tensile samples, indicating poor bonding with the concrete host matrix. However, the Zoltek carbon fiber and recycled carbon fiber showed reduced pullout and higher tensile strength, indicating improved bonding due to compatible sizing and rough surface morphology compared to the Hexcel carbon fiber.
6. Carbon fibers did not provide strength post-cracking, potentially due to the fiber shearing once the cracks developed.

In this study, it was observed using carbon fiber as a replacement for steel fiber has been shown to provide comparable or better mechanical performance, with recycled carbon fiber being of particular interest because it is potentially more economically feasible compared to other types of carbon fiber. One major challenge with using carbon fiber in concrete is the decrease in workability, which leads to increased void content, which was observed with both simple density measurements and micro X-ray computed tomography scans. The total void volume and void size both negatively affect the strength of the concrete, and, in some cases, can completely negate the benefits of carbon fiber. The fibers pulled out of the concrete without developing their full strength, showing that there was poor bonding between the fibers and the concrete, thus further reducing the benefit of the fiber. The effects of fiber bonding are shown to significantly improve the tensile strength of concrete, with the sized Zoltek carbon fibers showing particular promise in tension. Unfortunately, the Zoltek fibers also showed poor workability due to their length. In future studies, the workability and void content of carbon fiber-reinforced concrete should be carefully evaluated to ensure that the fibers being used can achieve their full potential, and how well the fibers bond within the concrete should be examined. Overall, it was demonstrated that recycled carbon fibers as a reinforcement in ultra-high performance are comparable with the commercially available aerospace-grade carbon fiber and intermediate low-cost carbon fibers, along with steel fiber reinforcement and neat concrete mixes used in this study. 

## Figures and Tables

**Table 1:** UHPC mix design for steel and carbon fibers in units of kg of material per cubic meter of concrete.

Materials	Steel Fiber	Carbon Fiber
	kg/m^3^	kg/m^3^
Ductal^®^ dark gray premix	2243	2243
Water	111	111
CHRYSO^®^Premia 150 high-range water reducer	31	31
Fibers	156	36

**Table 2:** Physical and mechanical properties of fibers used in UHPC mix designs.

Fiber Type	Notes	Density (g/cm^3^)	Tensile Strength (MPa)	Tensile Modulus (GPa)	Fiber Length (mm)	Diameter (μm)	Aspect Ratio
Steel fiber	ASTM A820 Type 1 [91]	7.8 [99]	345	200	13	200	65
Hexcel carbon fiber [98]	Mix of AS and IM	1.80	4447–6826	231–313	6.4	4.4–7.1	901–1454
Zoltek carbon fiber [79]	Sized	1.82	4137	242	12.7	7.2	1764
Recycled carbon fiber	-	1.81	-	-	1.5 ± 1.2	6.7 ± 0.8	224

Note: ± values are standard deviation.

**Table 3:** Thermogravimetric isothermal properties of carbon fibers in a nitrogen atmosphere.

Fiber Type	Total Degradation of Sizing Content (%)
Hexcel carbon fiber	5.91
Zoltek carbon fiber	0.75–2.54
Recycled carbon fiber	0.99

**Table 4:** Mechanical properties of different concrete mixes.

Mix	Flow (%)	Compression Cubes (MPa) *n* = 3	Tension (MPa) *n* = 3	Flexural (MPa)	Density (g/cm^3^) *n* = 2
Neat	133	87.9 (10)	5.21 (0.30)	11.7 (2.27) *n* = 2	2.49 (0.23)
Steel	74	113.1 (5.7)	6.58 (0.64)	12.9 (2.79) *n* = 3	2.42 (0.05)
Hexcel carbon fiber	44	134.5 (3.6)	5.29 (0.33)	10.8 (1.17) *n* = 2	2.17 (0.02)
Zoltek carbon fiber	27	107.2 (4.5)	8.99 (0.72)	9.83 (0.96) *n* = 4	2.23 (0.01)
Recycled carbon fiber	36	135.3 (8.4)	6.89 (0.70)	10.7 (1.04) *n* = 4	2.30 (0.04)

Note: The value in parentheses represents the standard deviation of each sample set, and *n* is the number of samples.

**Table 5:** Void volume fraction of concrete mix designs.

Sample Type	Void Volume Fraction (%)
Neat	3.46
Steel	2.55
Hexcel	4.02
Zoltek	4.38
rCF	3.98

## Data Availability

The data in this study are available upon reasonable request.
